# Supplementary material for: Implementing DASH-Aligned Meals and Self-Measured Blood Pressure to Reduce Hypertension at Senior Centers: A RE-AIM Analysis
Source: Nutrients. 2022 Nov 18;14(22):4890. doi: 10.3390/nu14224890 (PMC9699075; doi:10.3390/nu14224890)
Supplement: Supplementary file 1 [file nutrients-14-04890-s001.zip › nutrients-2000884-supplementary.pdf]

Table S1. Mission/Services of Advisory Committee Members.

Services/Populations Best Aligned with Organizational Mission (select all that apply)	% (n)
**Seniors (older adults)**	52.0% (13)
**Food Security/Scarcity**	28% (7)
**Nutrition**	36% (9)
**Health Services (hospital, healthcare system, vendor)**	28% (7)
**Wellness**	24% (6)
**Health Research**	56% (14)
**Health Policy**	16% (4)
**Populations Affected by Health Disparities**	20% (5)
